# Association of bone metabolic markers, bone mineral density and clinical characteristics with the occurrence of secondary fractures after PVP/PKP for osteoporotic thoracolumbar fractures: a study on logistic

**DOI:** 10.3389/fsurg.2026.1730811

**Published:** 2026-07-30

**Authors:** Jiawei Fu, Guanhua Xu, Jiajia Chen, Zhiming Cui

**Affiliations:** Department of Spine, Affiliated Nantong Clinical College of Nantong University, Nantong First People's Hospital, School of Medicine, Nantong University, Nantong, Jiangsu, China

**Keywords:** bone metabolism, bone mineral density, osteoporotic thoracolumbar fracture, percutaneous kyphoplasty, percutaneous vertebroplasty, secondary fracture

## Abstract

**Objective:**

To investigate the relationship between bone metabolism markers, bone mineral density (BMD), clinical characteristics, and the occurrence of secondary fractures after percutaneous vertebroplasty (PVP) or percutaneous kyphoplasty (PKP) in patients with osteoporotic thoracolumbar fractures (OTF).

**Methods:**

A retrospective study was conducted on 107 OTF patients treated in our hospital from May 2023 to May 2024. Patients were divided into secondary fracture and non-fracture groups. Standard univariate analysis, Pearson correlation, collinearity diagnostics, and multivariate logistic regression were performed to identify independent risk factors. A predictive nomogram was constructed and internally validated via bootstrap resampling.

**Results:**

Significant differences were observed between the fracture and non-fracture groups regarding age, number of operated vertebrae, incidence of cement leakage, levels of type I collagen amino-terminal propeptide (PINP), levels of 25-hydroxyvitamin D (25(OH)D), and BMD (all *P* < 0.05). Pearson correlation analysis confirmed that all these indicators were strongly correlated with secondary fracture occurrence. Multivariate logistic regression confirmed all six variables as independent risk factors. The nomogram constructed from these factors demonstrated excellent discriminative performance, with an area under the curve (AUC) value of 0.975. The calibration curve indicated no significant difference between predicted and actual probabilities, demonstrating high predictive value.

**Conclusion:**

Age, number of operated vertebrae, cement leakage, PINP, 25(OH)D, and BMD are closely associated with and are independent risk factors for secondary fractures after PVP/PKP in OTF patients. The nomogram model based on these factors has high value. Screening high-risk populations using these indicators provides crucial theoretical guidance for the clinical prevention and treatment of secondary fractures.

## 1 Introduction

Osteoporosis (OP) is a common disease in the elderly population, characterized by decreased bone mass and bone microstructural degradation. As China's population ages, the incidence of OP and the related compression fractures continues to increase annually, posing a significant threat to the health of elderly individuals and becoming a major public health issue (1–3). Luo et al. (4) reported that OP is the most common cause of compression fractures. As the core region of spinal biomechanical loading, the thoracolumbar spine has a high incidence of osteoporotic fractures, accounting for approximately 70% of all spinal fractures. OP fracture not only leads to severe pain, activity limitation and quality of life decline, but also may be accompanied by neurological impairment, spinal kyphosis, and other complications, which significantly increase the disability rate and mortality rate, and have a serious impact on the patient's physical health and life safety. It is of great significance to adopt scientific and effective treatment programs (5–7).

Percutaneous vertebroplasty (PVP) and percutaneous kyphoplasty (PKP), as representative minimally invasive procedures, are among the most important surgical treatments for osteoporotic thoracolumbar fractures (OTF). These techniques can rapidly restore the compressed vertebral body, alleviate patient pain, and achieve therapeutic goals (8, 9). However, with the popularization of surgical techniques, the problem of postoperative secondary fractures has gradually attracted attention. Studies have reported that the incidence of secondary fractures after PVP/PKP ranges from 10% to 30%, with the underlying mechanism involving synergistic effects among biomechanical alterations, bone metabolism imbalance, and patient-specific risk factors (10). Secondary fractures not only aggravate the physical and mental burden of patients, but also may affect the long-term efficacy of surgery and even lead to treatment failure. Therefore, exploring the risk factors of secondary fractures and establishing an effective prediction model have important clinical value for optimizing perioperative management and improving patient prognosis. However, few studies have been reported on the causes of secondary fractures after PVP/PKP, and they mainly focus on cement leakage, insufficient vertebral height restoration, and stress concentration in neighboring segments. Shevroja et al. (11) stated that bone mineral density (BMD), as the gold standard for osteoporosis diagnosis, has been proved to be closely related to the risk of fracture, however, its ability to predict postoperative secondary fractures has limitations. Limitations. The application of bone metabolic markers in osteoporosis diagnosis and treatment has attracted much attention, and by dynamically reflecting the balanced state of bone formation and bone resorption, it is able to more sensitively assess bone metabolic activity and the efficacy of anti-osteoporosis treatment (12, 13). In addition, the clinical characteristics of patients may indirectly increase the risk of postoperative secondary fractures by influencing bone metabolism or skeletal biomechanical properties. However, most of the existing studies are limited to single-factor analysis, failing to systematically integrate the multidimensional data of bone metabolism, bone mineral density, and clinical characteristics, resulting in insufficient accuracy of the postoperative secondary fracture risk prediction model.

Based on this, in this study, we investigated the relationship between bone metabolism markers, bone mineral density and clinical characteristics and the occurrence of secondary fracture after PVP/PKP, and constructed a multifactorial nomogram model of bone metabolism, bone mineral density and clinical characteristics, to provide a new perspective for identifying risk factors associated with secondary fractures after PVP/PKP, with the aim of identifying high-risk patients at an early stage, thereby improving the long-term outcome of the surgery and reducing the consumption of medical resources.

## 2 Materials and methods

### 2.1 Statement of ethics

This study was approved by the Institutional Review Board and Ethics Committee of the Institution. Given that this study was retrospective and only de-identified subject data were used, informed consent was not required as there was no risk or adverse effect on the subjects. This waiver is in accordance with regulations and ethical guidelines related to retrospective studies.

### 2.2 Study design

This retrospective analysis examined the clinical data of 107 OTF patients admitted to our hospital during the period of May 2023-May 2024 as study subjects. The data of the study subjects were obtained from the medical record system, and the patients were divided into two groups according to whether they had a secondary fracture after surgery: the group that did not occur (*n* = 82), and the group that did occur (*n* = 25).

### 2.3 Nano-exclusion criteria

Inclusion criteria; (1) preoperative full-spine anteroposterior radiographs and magnetic resonance imaging (MRI) of the fracture site confirming the diagnosis of OTF, with postoperative radiography reviewed immediately after surgery; (2) age >18 years; (3) complete clinical data; (4) surgery performed using either PVP or PKP alone; (5) diagnosis meeting the relevant criteria in the Clinician's Guide to Prevention and Treatment of Osteoporosis (14); (6) all patients meeting the surgical indications.

Exclusion criteria: (1) burst fracture; (2) intrusion of the bone mass into the spinal canal, causing neuromedullary symptoms and requiring decompression; (3) patients requiring open or percutaneous pedicle screw fixation; (4) allergy to bone cement and contrast media; (5) comorbidities with other spinal disorders, which resulted in significant changes in electrical impedance at the surgical site due to changes in bone pathology; and (6) comorbidities with neurologic disorders or cognitive dysfunction.

### 2.4 Surgical methods

After satisfactory anesthesia, patients were placed in the prone position with the abdomen suspended. For PVP surgery: C-arm fluoroscopy was used to identify the injured vertebrae and the lateral projection points of the bilateral pedicles using a localization plate, which were marked with a pen. Intraoperative electrocardiographic monitoring was performed throughout the procedure. Routine disinfection and laying of towels, skin for 0.5 cm incision, using the left 10 o'clock and right 2 o'clock pedicle approach, puncture needle tip pointing inward and downward, tilted about 25° fluoroscopy under the puncture needle slowly drilling, through the pedicle into the vertebral body, C-arm fluoroscopy orthotropic through the pedicle, lateral position is located in the middle and posterior 1/3 of the vertebral body. The puncture needle was withdrawn, the hole was reamed and a working trocar was placed along the guide pin to establish a working channel. Blend the bone cement (polymethylmethacrylate), under C-arm fluoroscopic monitoring, push and inject the appropriate amount of bone cement, see that the bone cement in the vertebral body is well filled, pull out the pusher and insert the puncture needle. After the bone cement was hardened, the sleeve and needle were withdrawn, and a sterile dressing was applied. Electro-permeability again to confirm that the bone cement is well filled in the vertebral body, the patient has no special discomfort, and returned to the ward. PKP surgery: after establishing the working channel, it is necessary to put in the balloon for expansion, put in the balloon expander to the anterior middle 1/3 of the vertebral body, gradually pressurize the vertebral body under the fluoroscopic view of the C-arm as much as possible to restore the height of the vertebral body, and then exit the expander, adjust the bone cement injection, and then wait until the bone cement is well-filled in the vertebral body, pull out the push rod and insert the puncture needle, and then wait until the bone cement is hardened, pull out the puncture needle, and then put on the aseptic dressings. After confirming that the bone cement was well filled in the vertebral body by electro-permeability again and the patient had no special discomfort, the patient returned to the ward.

### 2.5 Data collection

All patients underwent surgical treatment, and general demographic data of the study population were registered and collected, including: gender (male/female), age, body mass index, smoking history (previous smoking or currently still smoking, smoking ≥5 cigarettes/day, and smoking duration of more than 5 years) (yes/no), drinking history (defined as the number of drinking ≥3 times/week) (yes/no), hypertension [meeting the relevant criteria in the Chinese Guidelines for Prevention and Control of Hypertension (15) for relevant criteria] (yes/no), diabetes mellitus [meeting the diagnostic criteria in the Chinese Guidelines for Prevention and Control of Type 2 Diabetes Mellitus (16)] (yes/no), hyperlipidemia [meeting the diagnostic criteria in the Chinese Guidelines for Prevention and Control of Dyslipidemia in Adults (17) for diagnostic criteria] (yes/no), surgical procedure (PVP/PKP), vertebral body bone cement injections, number of vertebrae operated on (1/ ≥ 2) one), previous fracture history (yes/no), puncture method (unilateral/bilateral), and cement leakage (yes/no).

### 2.6 Bone metabolism markers

Fasting venous blood samples were collected in the morning. Serum was separated after centrifugation (model: LL900, manufacturer: Luoyang Hongshi Machinery Equipment Co., Ltd.) and stored at −70 °C. Electrochemiluminescence immunoassays were used to measure parathyroid hormone (PTH), PINP, and 25(OH)D levels.

### 2.7 BMD detection

Dual-energy x-ray bone densitometer (model: Dexa Pro-Ⅰ, manufacturer: Tianjin Shenghong Medical Devices Co., Ltd.) was used to detect the BMD of injured vertebrae in all study subjects.

### 2.8 Postoperative secondary fracture evaluation criteria

Postoperative patients all completed a 1-year follow-up, and if the patients again experienced low back pain during the follow-up period, which was suggested by x-ray examination of decreased vertebral body height and confirmed by magnetic resonance examination, they were diagnosed as secondary fracture after PVP/PKP. Patients were categorized into the occurrence and non-occurrence groups according to whether or not a secondary fracture occurred.

### 2.9 Observation indexes

1. Demographic and clinical characteristics: age, sex, body mass index (BMI), smoking history, alcohol consumption history, hypertension, diabetes mellitus, hyperlipidemia, surgical procedure (PVP/PKP), number of operated vertebrae, cement leakage, and prior fracture history.
2. Bone metabolic markers: serum levels of parathyroid hormone (PTH), type I collagen amino-terminal propeptide (PINP), and 25-hydroxyvitamin D (25(OH)D).
3. Bone mineral density (BMD): preoperative BMD of the fractured vertebra measured by dual-energy x-ray absorptiometry.

### 2.10 Statistical methods

In this study, SPSS 25.0 and R software (version 4.3.1, packages: car, rms, pROC) were used for statistical analysis. Categorical variables were presented as frequencies (*n*) and percentages, and between-group comparisons were performed using the *χ*^2^ test or Fisher's exact test as appropriate. Continuous variables conforming to a normal distribution were expressed as mean ± standard deviation, and the independent samples *t*-test was used for inter-group comparisons; non-normally distributed continuous variables were reported as median (interquartile range), with the Mann-Whitney *U* test for between-group comparisons. A two-tailed *P* < 0.05 was considered statistically significant. Univariate analysis was first conducted to screen potential risk factors for secondary fractures. Pearson correlation analysis was applied to assess the linear correlation between candidate continuous variables and secondary fracture occurrence, while point-biserial correlation was used for dichotomous variables. Subsequently, a univariate logistic regression analysis was conducted to assess whether each candidate variable [age, number of operated vertebrae, bone cement leakage, type I prealbumin, 25(OH)D, and bone density] affected the occurrence of secondary fractures. Before multivariate regression, collinearity diagnostics were performed using variance inflation factor (VIF) and tolerance values. Variables with VIF ≤ 10 and tolerance ≥0.1 were considered to have no significant multicollinearity and were eligible for inclusion in the regression model. Multivariate binary logistic regression analysis was performed to identify independent risk factors for secondary fractures after PVP/PKP. Variables were entered into the model using the enter method to ensure full adjustment for confounding effects of all preselected predictors. Odds ratios (OR) and their 95% confidence intervals (95% CI) were calculated to quantify the strength of association between each independent variable and secondary fracture risk. A predictive nomogram was constructed based on the final logistic regression model to visually quantify the individual risk of secondary fractures. The discriminative ability of the nomogram was evaluated using the receiver operating characteristic (ROC) curve, with the area under the curve (AUC) calculated; an AUC value closer to 1 indicates better discriminative performance.

## 3 Results

### 3.1 Unifactorial analysis affecting the occurrence of secondary fracture after PVP/PKP surgery in OTF patients

Comparisons between the occurrence and non-occurrence groups revealed statistically significant differences in age, number of operated vertebrae, cement leakage, PINP, 25(OH)D, and BMD (*x*^2^/*t*/*Z* = 4.601, 8.379, 7.396, 5.413, 5.534, 9.806), *P* < 0.05). However, there were no statistically significant differences in Sex, Body mass index, drinking history, hypertension, Diabetes, hyperlipemia, mode of operation, the amount of vertebral bone cement injected, number of operated vertebrae, history of fracture, puncture method, and PTH indicators (*P* > 0.05) Table 1.

**Table 1:** Univariate analysis of secondary fractures after PVP/PKP in patients with OTF.

Index	Genetic set (*n* = 25)	Nonoccurrence group (*n* = 82)	*x*^2^/*t*/*Z*	*P*
Sex (*n*, %)				
Male	18 (72.00)	50 (60.98)	1.005	0.316
Female	7 (28.00)	32 (39.02)		
Age (years, *x* ± *s*)	64.00 (53.00, 69.00)	48.00 (35.00, 54.00)	4.601	<0.001
Body mass index (kg/m^2^, *x* ± *s*)	22.81 ± 2.30	22.89 ± 2.32	0.151	0.880
Smoking history (*n*, %)				
Yes	12 (48.00)	35 (42.68)	0.220	0.639
No	13 (52.00)	47 (57.32)		
Drinking history (*n*, %)				
Yes	10 (40.00)	22 (26.83)	1.585	0.208
No	15 (60.00)	60 (73.12)		
Hypertension (*n*, %)				
Yes	4 (16.00)	7 (8.54)	1.157	0.282
No	21 (84.00)	75 (91.46)		
Diabetes (*n*, %)				
Yes	3 (12.00)	5 (6.10)	0.965	0.326
No	22 (88.00)	77 (93.90)		
Hyperlipemia (*n*, %)				
Yes	1 (4.00)	4 (4.88)	0.033	0.856
No	24 (96.00)	78 (95.12)		
Mode of operation (*n*, %)				
PVP	7 (28.00)	22 (26.83)	0.013	0.908
PKP	18 (72.00)	60 (73.17)		
The amount of vertebral bone cement injected (*n*, %)				
<5.5 mL	14 (56.00)	40 (48.78)	0.339	0.527
≥5.5 mL	11 (44.00)	42 (51.22)		
Number of operated vertebrae (*n*, %)				
1	9 (36.00%)	56 (68.29%)	8.379	0.004
≥2	16 (64.00%)	26 (31.71%)		
History of fracture (*n*, %)				
Yes	2 (8.00)	2 (2.44)	1.646	0.199
No	23 (92.00)	80 (97.56)		
Puncture method (*n*, %)				
Unilateral	15 (60.00)	40 (48.78)	0.965	0.326
Bilateral	10 (40.00)	42 (51.22)		
Cement leakage (*n*, %)				
Yes	5 (20.00)	3 (3.66)	7.396	0.007
No	20 (80.00)	79 (96.34)		
PTH (ng/mL, *x* ± *s*)	43.62 ± 6.05	44.76 ± 5.88	0.843	0.401
PINP (ng/L, *x* ± *s*)	88.65 ± 20.02	68.37 ± 15.16	5.413	<0.001
25 (OH) D (ng/L, *x* ± *s*)	16.09 ± 5.11	21.74 ± 4.26	5.534	<0.001
BMD (*n*, %)				
≥−2.85 g/cm^3^	6 (24.00)	49 (59.76)	9.806	0.002
<−2.85 g/cm^3^	19 (76.00)	33 (40.24)		

### 3.2 Correlation of each index with the occurrence of secondary fracture after PVP/PKP in OTF patients

As shown by Pearson's correlation analysis, age, number of operated vertebrae, cement leakage, PINP, and the categorization of low BMD (coded as 1 for BMD < −2.85 g/cm^3^ indicating lower bone mineral density, and 0 for BMD ≥ −2.85 g/cm^3^) were positively correlated with the occurrence of secondary fracture after PVP/PKP in OTF patients (*r* = 0.483, 0.280, 0.263, 0.467, 0.303, *P* < 0.05), and 25(OH)D was negatively correlated with the occurrence of secondary fractures after PVP/PKP in OTF patients (*r* = −0.475, *P* < 0.05), as shown in Table 2.

**Table 2:** Correlation between each index and secondary fracture after PVP/PKP in OTF patients.

Index	Secondary fractures occurred after PVP/PKP in OTF patients	
	*r*	*P*
Age	0.483	<0.001
Number of operated vertebrae	0.280	0.004
Cement leakage	0.263	0.006
PINP	0.467	<0.001
25(OH)D	−0.475	<0.001
BMD	0.303	0.002

### 3.3 Univariate logistic regression analysis affecting the occurrence of secondary fractures after PVP/PKP in OTF patients

Taking whether a secondary fracture occurred as the dependent variable (with values: 0 = no occurrence, 1 = occurrence), age, the number of surgical vertebrae, bone cement leakage, PINP, 25(OH)D, and BMD were used as independent variables, and a single-factor Logistic regression analysis was conducted separately. The results showed that age, the number of surgical vertebrae (≥2), bone cement leakage, PINP, and BMD (<−2.85 g/cm^3^) were risk factors for postoperative secondary fractures (OR > 1, *P* < 0.05), while 25(OH)D was a protective factor (OR < 1, *P* < 0.05). The specific results are shown in Table 3. All variables met the conditions for entering the multivariate Logistic regression (single-factor Logistic regression analysis *P* < 0.05).

**Table 3:** Results of univariate logistic regression analysis.

Influencing factor	*Β* value	SE	Wald *X*^2^	*P*	OR value	OR value 95% CI
Age	0.119	0.028	17.860	<0.001	1.126	1.066–1.190
Number of operated vertebrae	1.448	0.521	7.728	0.005	4.256	1.533–11.816
Cement leakage	1.980	0.751	6.950	0.008	7.243	1.661–31.592
PINP	0.083	0.020	16.492	<0.001	1.087	1.044–1.131
25(OH)D	−0.290	0.069	17.766	<0.001	0.748	0.654–0.857
BMD	1.735	0.518	11.207	0.001	5.671	2.054–15.659

### 3.4 Covariance analysis

All variables were analyzed for VIF covariance using the R language (R package: logreg6.2.0), and the VIF of each variable was ≤10, and the tolerance was ≥0.1, so there was no problem of covariance, and the above indexes could be included in the Logistic regression model, as shown in Table 4.

**Table 4:** Collinearity analysis.

Influencing factor	VIF value	Tolerance
Age	1.069	0.935
Number of operated vertebrae	3.343	0.299
Cement leakage	1.149	0.870
PINP	1.199	0.834
25(OH)D	1.152	0.868
BMD	3.197	0.313

### 3.5 Multifactorial logistic logistic regression analysis affecting the occurrence of secondary fractures after PVP/PKP in OTF patients

**Figure 1:**
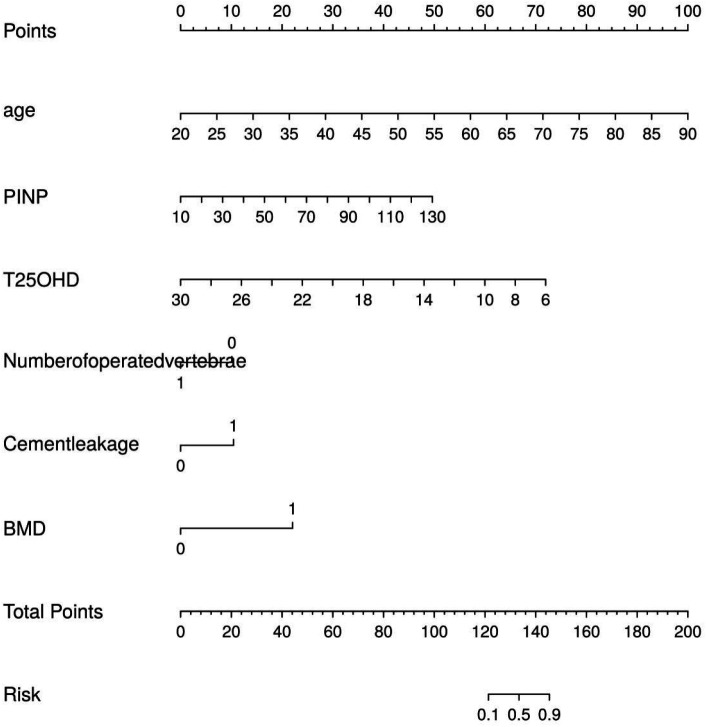
Nomogram prediction model.

As a result of the logistic regression analysis, it was found that age, the number of operated vertebrae, cement leakage, PINP, 25(OH)D, and BMD were all independent risk factors affecting the occurrence of secondary fractures after PVP/PKP in OTF patients (OR = 1.115, 3.829, 6.583, 1.084, 0.753, and 4.702, 95% CI = 1.059–1.175, 1.496–9.801, 1.450–29.894, 1.044–1.125, 0.660–0.859, and 1.698–13.019, *P* < 0.05), as shown in Table 5. According to the results of multifactorial logistic regression analysis, age, number of operated vertebrae, cement leakage, PINP, 25(OH)D, and BMD, a total of six independent risk factors were constructed to construct a nomogram model to visualize and individually assess the probability of occurrence of secondary fracture after PVP/PKP in patients with OTF, and the total of the prediction point scores corresponding to each variable corresponded to the bottom scale of the nomogram model, as shown in Figure 1.

**Table 5:** Multivariate logistic logistic regression analysis of secondary fractures after PVP/PKP in patients with OTF.

Influencing factor	*Β* value	SE	Wald *X*^2^	*P*	OR value	OR value 95% CI
Age	0.109	0.026	17.027	<0.001	1.115	1.059–1.175
Number of operated vertebrae	1.343	0.480	7.840	0.005	3.829	1.496–9.801
Cement leakage	1.885	0.772	5.959	0.015	6.583	1.450–29.894
PINP	0.080	0.019	17.460	<0.001	1.084	1.044–1.125
25(OH)D	−0.284	0.067	17.780	<0.001	0.753	0.660–0.859
BMD	1.548	0.520	8.875	0.003	4.702	1.698–13.019

### 3.6 Validation curve analysis

**Figure 2:**
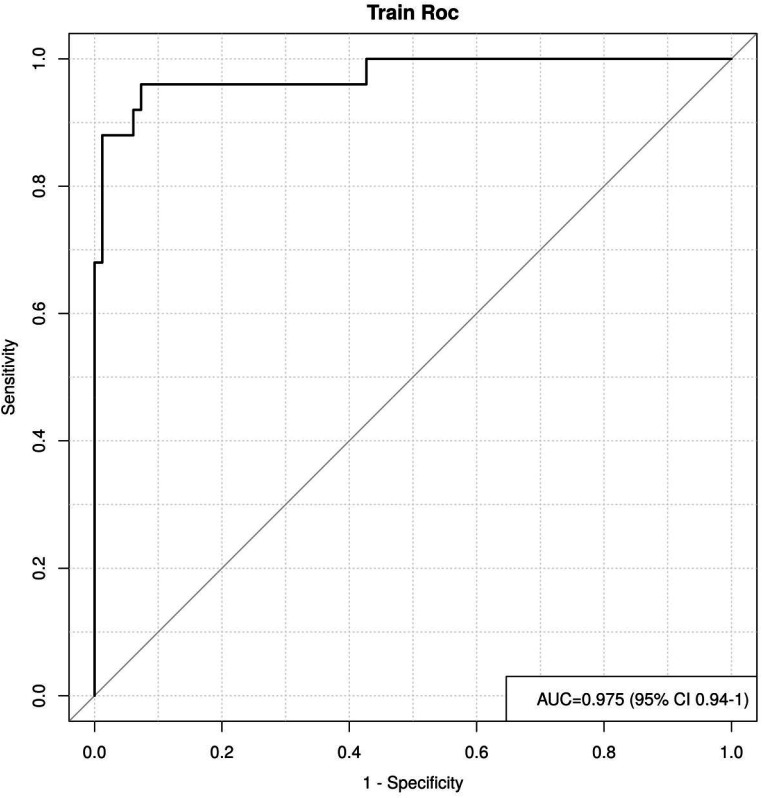
Calibration curve.

The calibration and reference curves of the risk prediction model showed high differentiation and calibration, and demonstrated that the difference between the predicted probability and the actual probability was not statistically significant by the AUC value of 0.975, proving the high agreement between the actual and the predicted secondary fracture of the PVP/PKP postoperative period of the patients with OTF, as shown in Figure 2.

## 4 Discussion

PVP and PKP are widely used in OTF treatment because of their minimally invasive and rapid pain relief characteristics, and both of them restore the original biomechanics of the injured vertebrae by injecting bone cement into the vertebral body to correct the deformity and return it to normal (18, 19). However, referring to the study of Wang et al. (20), it is known that some patients have secondary fractures after receiving PVP and PKP treatments, which seriously threatens the patients' health and life safety. Therefore, actively analyzing the relevant factors affecting the occurrence of postoperative secondary fractures is the key to taking appropriate preventive and curative measures to improve clinical prognosis.

PINP mainly reflects the rate of type I collagen synthesis and bone conversion, which is a specific and sensitive indicator of new bone formation and the preferred bone formation marker; 25(OH)D is produced by the conversion of vitamin D through hydroxylation in the liver, which can reflect the level of vitamin D storage in the human body; BMD is a key indicator of bone strength, measured by dual-energy x-ray absorptiometry, and reflects the mineral content and density of the human skeleton (21, 22). It can be used to predict the likelihood of fracture in patients (21, 22). The data in this study showed that the comparison of PINP, 25(OH)D, and BMD between the occurrence group and the non-occurrence group showed significant differences, with positive correlation between PINP and BMD and postoperative secondary fractures after PVP/PKP, and negative correlation between 25(OH)D and postoperative secondary fractures after PVP/PKP, and all of them were independent risk factors affecting the occurrence of postoperative secondary fracture (*P* < 0.05). A possible interpretation of this association is that PINP, as a bone formation marker, reflects the status of bone metabolism. Higher PINP levels may indicate an active bone turnover state, which has been previously linked to increased fracture risk in osteoporotic populations (23). Although the present study cannot establish causality, this observed association suggests that patients with elevated PINP levels might have underlying metabolic imbalances that predispose them to secondary fractures. Vitamin D is involved in the process of bone metabolism and is closely related to muscle strength. Serum 25(OH)D is the way of storage and transportation of vitamin D in the body, and its level can fully reflect the nutritional status of vitamin D, which has the function of regulating the activity of osteoblasts, and then affects the health of the bones, which is similar to the argument of Chen et al. (24). And referring to previous studies, it can be seen that 25(OH)D can open calcium channels through the signaling system, exert its rapid regulation of calcium and phosphorus metabolism, promote the absorption of calcium and phosphorus in the small intestine, and affect the calcium metabolism of bone tissues, so as to maintain the normal level of blood calcium and blood phosphorus, and to promote osteocalcinosis (25). Low 25(OH)D levels have been previously associated with impaired calcium metabolism and increased bone resorption in experimental and clinical studies (25, 26) Our finding that lower 25(OH)D fractures is consistent with these established relationships, suggesting that vitamin D status may serve as a useful predictor of postoperative fracture risk. According to Wang et al. (26), BMD is an important indicator of bone quality that reflects the severity of osteoporosis. Low BMD indicates more severe osteoporosis and a higher likelihood of trabecular fracture and vertebral re-fracture when the spine is subjected to external forces after PVP/PKP. BMD is the core indicator of evaluating the strength of bone, and low BMD suggests that trabeculae are sparse, the cortex of the bone becomes thinner, and microstructures are fractured, which results in a significant reduction in the vertebral body's compressive strength. Even if the height of the vertebral body is partially restored after surgery, low BMD will easily lead to an imbalance of bone resorption-bone formation coupling due to stress concentration, accelerated bone loss due to enhanced osteoclast activity, and further weakening of bone repair ability due to insufficient compensation of bone formation, resulting in a vicious cycle of “low bone mass-fracture-re-fracture”. Thus, reduced 25(OH)D levels may exacerbate the high bone turnover state and weaken bone mineralization capacity by activating the PTH pathway. BMD and PINP together reflect the dual risk of “low bone strength and bone metabolism disorder,” potentially forming a vicious cycle of accelerated bone loss and declining bone quality. Therefore, comprehensive interventions before PVP/PKP treatment in OTF patients should take into account bone metabolism regulation, mechanical environment optimization and nutritional support.

Through the data of this study, it was shown that the significant difference in the comparison of age, number of operated vertebrae, and cement leakage between patients in the occurrence group and the non-occurrence group were positively correlated with the secondary fracture after PVP/PKP in OTF patients, and belonged to the independent risk factors affecting the secondary fracture after PVP/PKP in OTF patients (*P* < 0.05), which was similar to the argument of Katayanagi et al. (27). Several factors may explain this association. First, aging reduces calcium intake and increases calcium excretion, leading to secondary hyperparathyroidism, decreased bone calcium content, and reduced bone mass. Second, elderly patients experience decreased sex hormone production and increased oxidative stress, which inhibits osteoblasts while promoting osteoclast activity, thereby exacerbating osteoporosis. Third, reduced physical activity and paraspinal muscle degeneration further weaken the protective capacity of the thoracolumbar spine, increasing the likelihood of postoperative secondary vertebral fractures. With reference to previous studies, it can be seen that elderly patients have significantly lower bone density, decreased bone remodeling ability, weakened osteoclast activity, accelerated disc degeneration, decreased spinal elasticity, decreased overall mechanical properties of the spine, and increased compensatory loads in neighboring segments after reinforcement of the vertebral body with cement, which makes postoperative spinal stability easier to be undermined, and is very likely to cause postoperative secondary stress fractures (28). The high number of operated vertebrae increases the risk of postoperative secondary fracture in patients may be related to the fact that patients with multisegmental vertebral fracture generally have more severe osteoporosis. Furukawa et al. (29) indicated that, on one hand, multivertebral fractures reflect more severe osteoporosis in patients, with extensive destruction of the trabecular bone structure and further reduction in the compressive strength of the remaining vertebrae after surgery. On the other hand, multivertebral surgery disrupts the overall line of force of the spine, and the local stiffness increases significantly after cement reinforcement, which makes cement injection more difficult and increases the possibility of uneven distribution, leading to an increase in the compensatory load of neighboring vertebrae, accelerating the accumulation of microinjuries in the unoperated vertebrae, and more significant local stress concentration, resulting in a higher risk of secondary fracture after surgery. Multi-vertebral surgery requires cement reinforcement of multiple vertebral bodies, and unreinforced vertebral bodies may be subjected to greater biologic stresses, causing changes in the local sagittal balance of the spine, which may lead to re-fracture of the vertebral body. Leakage of bone cement into the intervertebral disc or paravertebral vein can change the spinal stress distribution, leading to asymmetric load transfer, triggering disc hardening, loss of elasticity, destroying the spinal stress cushioning function, and altering the overall stress distribution in the spine, and the neighboring vertebrae are required to bear more mechanical loads by compensation, which results in the neighboring vertebrae being subjected to abnormal stress concentration and significantly increases the risk of re-fracture, which is further supported by Chen et al. The argument of Chen et al. (30) is further supported. It is also known from previous studies that leaking bone cement may stimulate the surrounding tissues to trigger aseptic inflammation, inhibit osteoblast differentiation, and promote osteoclast activity, leading to insufficient new bone formation, accelerating local bone resorption and bone loss, further weakening vertebral body strength, destroying vertebral plate structural stability, and leading to the expansion of localized microfractures, increasing the risk of secondary fractures. These findings suggest that age, number of operated vertebrae, and cement leakage interact through the dual pathways of ‘metabolism and mechanics,’ exacerbating spinal instability and bone quality deterioration, ultimately increasing the risk of secondary fractures after PVP/PKP. Therefore, clinical efforts should focus on reducing re-fracture rates through refined surgical techniques, rigorous anti-osteoporosis treatment, and long-term follow-up.

In this study, a nomogram prediction model was constructed by combining various factors, which transformed complex multifactorial regression equations into intuitive graphs for visual and individualized assessment of secondary fracture risk. The model yielded an AUC of 0.975, indicating excellent discriminative performance in this cohort. However, we fully recognize that this high AUC value must be interpreted with great caution. The present study is limited by its relatively small sample size, retrospective design, and single-center nature, and the dataset was not formally divided into independent training and validation sets, which may lead to overoptimistic performance estimates and potential overfitting. Notably, the high predictive accuracy likely stems from the strong and consistent associations of the selected variables [age, number of operated vertebrae, cement leakage, PINP, 25(OH)D, and BMD] with secondary fracture risk in our cohort, rather than inherent generalizability to other populations. Therefore, this model should be regarded as an exploratory tool for risk stratification in similar clinical settings, and its clinical application requires further rigorous validation in larger, multicenter, prospective cohorts with external independent datasets to minimize overfitting bias and confirm its generalizability.

The shortcomings of this study are: (1) The study is a small-sample, single-center study with a small sample size and limited to a single hospital, which may lead to insufficient generalizability and representativeness of the results, which is affected by factors such as geographic location, medical resources and patient selection, and may not be able to comprehensively reflect the situation in different regions or under different medical conditions. (2) Retrospective studies usually rely on previous data, which may have the problem of incomplete or missing data, and the studies do not mention whether the patients are followed up for a long period of time, which restricts the comprehensive assessment of disease progression. (3) The sample size of this study is limited, and it is a single-center retrospective study. The model has not yet undergone external independent validation, and its extrapolation is limited. Therefore, the clinical promotion value of this model still needs to be further verified. Currently, it can only be used as a clinical auxiliary assessment tool. Its application should be comprehensively judged in combination with the individual patient's condition and clinical practice. In the future, multi-center, large-sample prospective studies should be conducted and external groups should be included for verification to further improve the model performance and enhance its clinical applicability.

## 5 Conclusion

In summary, age, number of operated vertebrae, cement leakage, PINP, 25(OH)D, and BMD are variables associated with the occurrence of secondary fractures after PVP/PKP in OTF patients. A predictive model based on these factors demonstrated good discriminative ability in this cohort. These findings may assist clinicians in identifying high-risk patients for closer monitoring and preventive interventions.

## Data Availability

The original contributions presented in the study are included in the article/Supplementary Material, further inquiries can be directed to the corresponding author.
